# Anæsthetics

**Published:** 1872-01

**Authors:** 


ANÆSTHETICS.—Dr. S. R. Naisley (*Journal Mat. Med.*) says: "I have been in the habit of using the Bisulphide of carbon as a local anæsthetic for several years. I have tested its efficacy and potency in facial neuralgia, hemicrania, odontalgia and lumbago, and the relief it afforded to the sufferer was almost instantaneous. My mode of application was this: place a pledget of cotton into a small-mouth bottle, saturate it well with the Bisulphide and apply it to the painful part, and as soon as the patient complains of smarting sensation, change the bottle, carefully following the course of the principal nerve that seems to be involved in the difficulty. I have used a combination of Rhigolene and the oil of Peppermint as a local anæsthetic in a number of neuralgia cases, that presented themselves at my office for relief, and thus far my success in those cases has been far beyond my most sanguine expectations. After several applications they express themselves cured. I have recently been in the habit of adding an etheral collodion to the compound, and I am gratified to say that in the combination I have a specific which will, under almost any circumstances, when the part is accessible, relieve the patient instantaneously; its effects are magical. In a severe case of tic douloureux, where the famous 'Wolcott Pain Paint' failed to give the patient a moment's relief, this compound was tried, and in several minutes the patient was relieved, and expressed herself free from any pain and the apprehension of a recurrence of the fearful and terrible monster."

